# Genome-Wide Transcriptome and Expression Profile Analysis of *Phalaenopsis* during Explant Browning

**DOI:** 10.1371/journal.pone.0123356

**Published:** 2015-04-14

**Authors:** Chuanjun Xu, Biyu Zeng, Junmei Huang, Wen Huang, Yumei Liu

**Affiliations:** 1 Fujian Key Laboratory of Physiology and Biochemistry for Subtropical Plants, Fujian Institute of Subtropical Botany, Xiamen, 361006, P. R. China; 2 Xiamen Overseas Chinese Subtropical Plant Introduction Garden, Fujian Institute of Subtropical Botany, Xiamen, 361002, P. R. China; The National Orchid Conservation Center of China; The Orchid Conservation & Research Center of Shenzhen, CHINA

## Abstract

**Background:**

Explant browning presents a major problem for *in vitro* culture, and can lead to the death of the explant and failure of regeneration. Considerable work has examined the physiological mechanisms underlying *Phalaenopsis* leaf explant browning, but the molecular mechanisms of browning remain elusive. In this study, we used whole genome RNA sequencing to examine *Phalaenopsis* leaf explant browning at genome-wide level.

**Methodology/Principal Findings:**

We first used Illumina high-throughput technology to sequence the transcriptome of *Phalaenopsis* and then performed *de novo* transcriptome assembly. We assembled 79,434,350 clean reads into 31,708 isogenes and generated 26,565 annotated unigenes. We assigned Gene Ontology (GO) terms, Kyoto Encyclopedia of Genes and Genomes (KEGG) annotations, and potential Pfam domains to each transcript. Using the transcriptome data as a reference, we next analyzed the differential gene expression of explants cultured for 0, 3, and 6 d, respectively. We then identified differentially expressed genes (DEGs) before and after *Phalaenopsis* explant browning. We also performed GO, KEGG functional enrichment and Pfam analysis of all DEGs. Finally, we selected 11 genes for quantitative real-time PCR (qPCR) analysis to confirm the expression profile analysis.

**Conclusions/Significance:**

Here, we report the first comprehensive analysis of transcriptome and expression profiles during *Phalaenopsis* explant browning. Our results suggest that *Phalaenopsis* explant browning may be due in part to gene expression changes that affect the secondary metabolism, such as: phenylpropanoid pathway and flavonoid biosynthesis. Genes involved in photosynthesis and ATPase activity have been found to be changed at transcription level; these changes may perturb energy metabolism and thus lead to the decay of plant cells and tissues. This study provides comprehensive gene expression data for *Phalaenopsis* browning. Our data constitute an important resource for further functional studies to prevent explant browning.

## Background

Plant tissue culture is an important tool in both basic and applied studies as well as in commercial application, Such as for propagation of orchids, especially in production of *Phalaenopsis*. The *Phalaenopsis* genus of popular ornamental plants belongs to the family *Orchidaceae* and is mainly used in tissue culture because of its rapid propagation. However, explant browning in *Phalaenopsis* tissue culture presents a major problem in producing regenerated *Phalaenopsis* in culture. To date, little is known about the browning mechanisms of *Phalaenopsis*. Therefore, establishing effective and economical methods to reduce explant browning in tissue culture of *Phalaenopsis* will provide a valuable tool for plant propagation and transgenic manipulation.

Enzymatic oxidation of phenols produces tissue browning [1–3], which involves peroxidase (POD, EC 1.11.1.7), polyphenol oxidase (PPO, EC 1.10.3.1 or EC 1.14.18.1), and phenylalanine ammonia-lyase (PAL, EC 4.3.1.5) [3–7]. There are three hypotheses considered to be responsible for the mechanism of enzymatic browning, namely, phenol-phenolic enzyme regional distribution [8], free radical damage [9], and protective enzyme system [10]. Therefore, these browning enzymes are the important factors and necessary conditions for browning. Our previous studies demonstrated that PAL, PPO, and POD activities increase during browning of *Phalaenopsis* explants, and *PAL* and *POD* transcript levels consistently increase after 3 d of tissue culture [4, 5, 11]. Proteomic studies showed that peroxiredoxin, mitochondrial F-1-ATPase subunit 2, and regulatory protein-like protein increase in *Phalaenopsis* explants after 3 d of culture [12]. These findings clearly showed that browning of *Phalaenopsis* explants is tightly regulated at the transcriptome and proteome levels. These studies indicate that simply manipulating the activity of specific enzymes such as PPO may not solve the explant browning problem [13].

Recent advances in sequencing technologies have enabled genomic-scale sequencing projects for many model organisms. These projects include *de novo* transcriptome analysis and reference mapping of expressed transcripts [14–16]. Using RNA- sequencing (RNA-seq) techniques, Mellidou *et al* [17] identified different expressed gene (DEGs) mainly involved in lipid metabolism, secondary metabolism, and cell wall modifications in apple fruit browning disorder. The energy-related and stress-related genes were also altered during apple fruit browning development. To investigate pear fruit surface brown (SBS), transcriptome analysis showed that up-regulated the expression of genes related to oxidative phosphorylation, phenolic compound synthesis and PPO [18]. These studies provide a genomics basis for botanists to understand the molecular mechanisms of enzymatic browning.

Recently, OrchidBase (http://lab.fhes.tn.edu.tw/est) has been established from 37,979,342 sequence reads collected from 11 in-house *Phalaenopsis* orchid cDNA libraries using multiple sequencing techniques [19]. A total of 1,233,823 unique sequences were obtained from *Phalaenopsis aphrodite* using Roche 454 and Illumina/Solexa high-throughput sequencing technologies [20]. Based on these accomplishments in *Phalaenopsis* genome research, in the current study, we carried out transcriptome analysis and expression profiling of *Phalaenopsis* leaf explants during browning.

Here, we used Illumina short-read sequencing for *de novo* transcriptome assembly and analysis of *Phanalenopsis* hybrid: Konggangjinli (A red flower cultivar, S1 Fig) explants at the early stage of browning. We constructed a mixed library from 0-d-cultured (control), 3-d-cultured (prior to browning, browning rate 0%), and 6-d-cultured explant (brown rate 100%) (S2 Fig). After sequencing, we used BLAST to compare these reads to the NCBI database and OrchidBase (http://lab.fhes.tn.edu.tw/est) to determine their encoded proteins. The genes and isoforms were annotated and functionally mapped to Gene Ontology (GO) terms. Furthermore, we constructed 3 libraries from the above-mentioned three kinds of explant for expression profile analysis during *Phalaenopsis* explant browning. The reads were first mapped to the transcriptome and OrchidBase and then analyzed to identify differentially expressed genes (DEGs) among 0 d, 3 d and 6 d. DEGs were categorized into three groups: explants cultured for 3 d *vs*. 0 d (before explant browning), explants cultured for 6 d *vs*. 0 d (after explant browning), and explants cultured for 6 d *vs*. 3 d. Next, we confirmed the differential expressions of interesting genes by quantitative real-time polymerase chain reaction (qRT-PCR). This study sheds fresh light on explant browning at a genome-wide scale and will facilitate the functional analysis of browning-related genes in *Phalaenopsis*.

## Materials and Methods

### Plant material

Leaves of *Phalaenopsis sp*. plants were cut into 0.5 cm × 0.5 cm segments and cultured on Murashige & Skoog medium (Murashige & Skoog, 1962) solidified with 0.8% agar (w/v) at pH 5.8 and supplied with 3 mg/L 6-BA. All cultures were maintained under cool white fluorescent light (approximately 35 μmol m^–2^ s^–1^ photon flux density and 16 h photoperiod) at 24 ± 2°C. Explants were collected from leaves cultured for 0, 3, and 6 d, respectively. All explants were immediately frozen in liquid nitrogen and stored at -80°C.

### RNA isolation and Illumina PE library preparation for transcriptome analysis

*De novo* assembly of the transcriptome was performed by short-read sequencing (Illumina). Total RNAs were isolated from explants that were cultured for 0, 3, and 6 d, and then mixed for cDNA library construction. TRIzol Reagent (Invitrogen, CA, USA) was used to isolate total RNA following the manufacturer’s protocol. RNA quality was assessed using the Agilent 2100 Bioanalyzer with 260/280 and 260/230 absorbance ratios.

In this study, transcriptome libraries were constructed by Shanghai Majorbio Bio-pharm Biotechnology Co., Ltd. (Shanghai, China). The Truseq RNA sample prep kit (Illumina, San Diego, CA, USA) was used to construct the cDNA library according to the manufacturer’s protocol. Subsequently, the insert size of the libraries was selected for cDNA target fragments of 300 bp to 500 bp on 2% Low-range Ultra Agarose, followed by PCR amplification using Phusion DNA polymerase (NEB) for 15 PCR cycles. After being quantified by TBS380, libraries were paired-end sequenced using Illumina HiSeq 2000 (2 × 100 bp read length).

### Bioinformatics analysis of *Phalaenopsis* transcriptome

Prior to analyses, all adapter nucleotides were trimmed and the sequences were de novo assembled using SeqPrep, condetri v 2.0.pl and Trinity software (http://trinitynaseq.sourceforge.net) with default parameters respectively. We then collected equestris of *Phalaenopsis* from the OrchidBase (http://lab.fhes.tn.edu.tw/est.), which contained 37,979,342 sequence reads, to improve the assembly quality. Finaly, BLASTp /BLASTx alignment (E- value of ≤10^-5^) was performed between isogenes and protein databases such as NCBI’s non-redundant (nr), string. Homologous protein domains from translated transcriptomic sequences of *Phalaenopsis* were identified by searching against the Pfam database using Hmmscan (http://pfam.sanger.ac.uk/) [21]. BLAST2GO was used to assign putative functionalities, GO terms, and KEGG (Kyoto Encyclopedia of Genes and Genomes) based metabolic pathways [22]. Furthermore we conducted sequence similarity analysis of protein sequences between the assembled transcripts and *Arabidopsis thaliana*, *Nicotiana tabacum*, *Glycine max*, *Lycopersicon esculentum*, *Triticum aestivum*, *Oryza sativa*, and equestris of the *Phalaenopsis* database. In this analysis, the *Phalaenopsis* equestris database was downloaded from the NCBI database (http://www.ncbi.nlm.nih.gov/bioproject/53345), by BLASTn search with a cut-off (E- value of ≤10^-5^). Arabidopsis and Oryza were chosen as they represent dicot and monocot model species, respectively.

### Illumina PE library preparation for expression profile analysis and transcriptome mapping for bioinformatics analysis

To examine expression profiles, we then constructed and sequenced three independent libraries from 0 d, 3 d, and 6 d explants. Each cDNA library was constructed as described above and then sequenced by Illumina HiSeq 2000 (1 × 50 bp read length). After removing adaptor sequences, empty reads, and low-quality sequences with unknown sequences “N”, the high quality clean reads were mapped to our *Phalaenopsis* transcriptome reference database using the Bowtie software with default parameters. Then the transcript abundances were quantified by SEM (http://dewelabbiostat.wise.edu/rsem). The differentially expressed genes (DEGs) between the three samples were identified using the number of mapped reads as EdgeR inputs (http://bioconductor.org/packages/release/bioc/html/edgR.html). In this algorithm, we used FDR (False Discovery Rate) ≤0.05 and the absolute value of |log2FC|>1 as the threshold to judge the significance of differential gene expression. Here, FDR was used to determine the threshold p-value in multiple tests and analysis through manipulating the FDR value, where p-value corresponded to the differential gene expression test.

Three comparisons were conducted: explants cultured for 3 d *vs*. explants cultured for 0 d (3 d *vs*. 0 d), explants cultured for 6 d *vs*. explants cultured for 0 d (6 d *vs*. 0 d), and explants cultured for 6 d vs. explants cultured for 3 d (6 d vs. 3d). A Venn diagram was constructed using the Venny tool software [23].

The three groups of DEGs were mapped to GO terms. The DEGs were aligned to the GO database for GO functional enrichment analysis using GO tools software with FDR corrections (http://githb.com/tanghaibao/goatools). Statistical significance was considered at P<0.05. We also mapped all DEGs to the KEGG database and searched for significantly enriched KEEG pathways at P<0.05 level using KOBAS software (http://kobas.cbi.pku.edu.cn/home.do). The sequence similarity analysis of protein sequences between the DEGs with *Arabidopsis thaliana* and *Oryza sativa* were also conducted as mentioned above. Homologous protein domains for DEGs were identified by searching against the Pfam database using Hmmscan

### Validation of gene expression by quantitative real-time PCR (qRT-PCR)

qRT-PCR was performed to confirm the RNA-seq data. Total RNA was extracted for cDNA preparation as described above. Total RNA (1 μg) was reverse transcribed into single-stranded cDNA using the Primescript RT reagent kit (TaKaRa, Dalian, China). qRT-PCR was implemented using the SYBR premix Ex Taq kit (TaKaRa, Dalian, China), with first-strand cDNA as the template. The actin gene from *Phalaenopsis* was used as an internal control. The relative quantitative method (2^-ΔΔCt^) was used to calculate the fold change of the target genes. The primers employed in the qRT-PCR are listed in S1 Table. The results of these reference genes were compared to fragments per kilobase of exon model per millions mapped reads (FPKM) estimates.

### Date availability

The filtered reads for *Phalaenopsis spp* were deposited in the NCBI Sequence Read Archive (SRA) under the accession number SRX386023 and SRS506991 (http://www.ncbi.nlm.nih.gov/sra/?term=SRS506991, http://www.ncbi.nlm.nih.gov/sra/?term=SRX386023) (data unpublic)

## Results

### *Phalaenopsis* transcriptome assembly and annotation

To examine the *Phalaenopsis* transcriptome, we collected RNA from 0-d-cultured, 3-d-cultured, and 6-d-cultured explants and constructed one mixed cDNA library. We used the tagged cDNA library for 2×100 bp paired-end sequencing on a single lane of the Illumina Hiseq2000 and generated 118,996,000 raw paired-end reads resulting in 12 gigabases of sequence. After cleaning and quality control, this resulted in 79,434,350 reads (7,544,398,171 bp) (Table 1). Using the RNA seq *de novo* assembler Trinity, we obtained 31,708 isogenes with a mean size of 1108.07 bp and lengths from 351 bp to 9,265 bp (S2 Table). Using BLASTn sequence similarity search with cut-off E-value≤10^-5^, about 90% of isogenes (28,267/31,708) have significant sequence similarity to the *Phalaenopsis* EST database (data not shown). Only 10% of isogenes (3441) were not hit, and were thought to be specific sequence in our study. We used this reference transcriptome for our subsequent analysis of expression profiles during explant browning.

**Table 1:** Assembly of transcriptome of *Phalaenopsis*.

	Number
Total number of raw reads	118,996,000/12,018,596,000(bp)
Total number of reads assembled	79, 434, 350/7 544, 398, 171(bp)
Total genes	21, 348
Total isogenes	31, 708
Total residues	35, 134, 614
Smallest isogene	351
Largest iosgene	9, 265
average	1, 108.07

About 84% assembled transcripts (26,565/31,708, S3 Table) were annotated by using BLASTp and BLASTn algorithms to search against the NR, gene, string and Nt databases (E-value≤10-5). A BLASTx sequence similarity search with cut-off E-value≤10^-5^, showed that *Phalaenopsis* proteins have significant sequence similarity to the proteomes of other plants (Table 2). For example the *Phalaenopsis* proteome showed substantial amino acid sequence identity to *Arabidopsis thaliana* (61.71%) and *Oryza sativa* (63.78%) proteomes. The function of the *Phalaenopsis* sequences hit in *Arabidopsis thaliana* and *Oryza sativa* were shown as supporting information in S4 and S5 Tables. Most of the transcripts had most-significant hits to the *Phalaenopsis* equestris database, and only 14.34% of the sequences did not show significant identity in the *Phalaenopsis* equestris database. These do not found homologous sequences suggesting that they may novel proteins in our study.

**Table 2:** Homology analysis of *Phalaenopsis* proteome with other plants.

	Proteome of *Phalaenopsis*	Sequences number(identity≧30)	Sequences number(identity≧70)	Sequences number(identity≧80)	Average Sequences identity(≧30)
Arabidopsis thaliana	26565	1120(4.22%)	350(1.32%)	121(0.46%)	61.71%
Nicotiana tabacum	26565	2304(8.67%)	688(2.59%)	253(0.95%)	60.96%
Glycine max	26565	4386(16.51%)	1555(5.85%)	639(2.41%)	63.45%
Lycopersicon esculentum	26565	2237(8.42%)	731(2.75%)	267(1.01%)	62.35%
Triticum aestivum	26565	1197(4.51)	382(1.44%)	142(0.53%)	61.37%
Oryza sativa	26565	4735(17.82%)	1734(6.53%)	631(0.24%)	63.78%
*Phalaenopsis* equestris	26565	23050(86.78%)	17942(67.54%)	16951(63.81%)	85.66%

### *Phalaenopsis* expression profile changes during explant browning

To examine the expression of *Phalaenopsis* genes in browning, we next constructed three cDNA libraries and used 2×100 bp paired-end sequencing to obtain sets of expression reads for explants cultured for 0, 3, and 6 d. After removing the adaptor sequences and low-quality sequences, we mapped the clean reads for each sample (0, 3, and 6 d) to the assembled reference transcriptome of *Phalaenopsis* (Table 3).

**Table 3:** Assembly of *Phalaenopsis* expression profiles and mapping to transcriptome.

Sample	Number of sequences	Bases (bp)	Number of sequences mapped to transcriptome	Ratio of sequences hit in transcriptome	Total number ofgenes expressed
0 d	7, 750, 687	741, 453, 474	5, 405, 788/6, 249, 646	86.50%	19,156
3 d	9, 085, 070	868, 764, 389	5, 560, 155/7, 379, 172	75.35%	22,185
6 d	9, 529, 153	912, 039, 691	5, 841, 384/7, 755, 354	75.32%	22,902

We used Bowtie (http://bowtie-bio.sourceforge.net/index.shtml) to map reads, used RSEM to assign reads to genes and isoforms, and used edgeR to standardize the reads. We refer to the normalized reads in fragments per kilobase of exon model per millions mapped reads (FPKM), and we used the number of genes with FPKM values ≥0.5 to estimate the total number of genes expressed during *Phalaenopsis* explant browning(S6 Table). We found 19,156 expressed genes in the explants cultured for 0 d, representing almost 60% of the annotated transcriptome. We also found 22,185 genes (70%) were expressed in 3 d, and 22,902 (72%) in 6 d (Table 3).

### Identification of genes differentially expressed during *Phalaenopsis* explant browning

**Fig 1:**
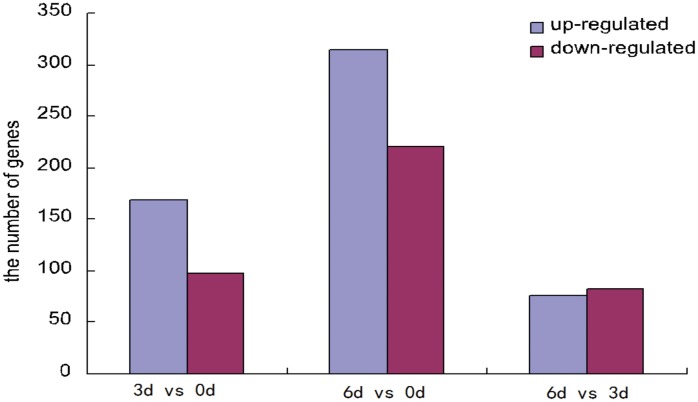
The number of up(blue)- and down(red)- regulate genes in three groups.

**Fig 2:**
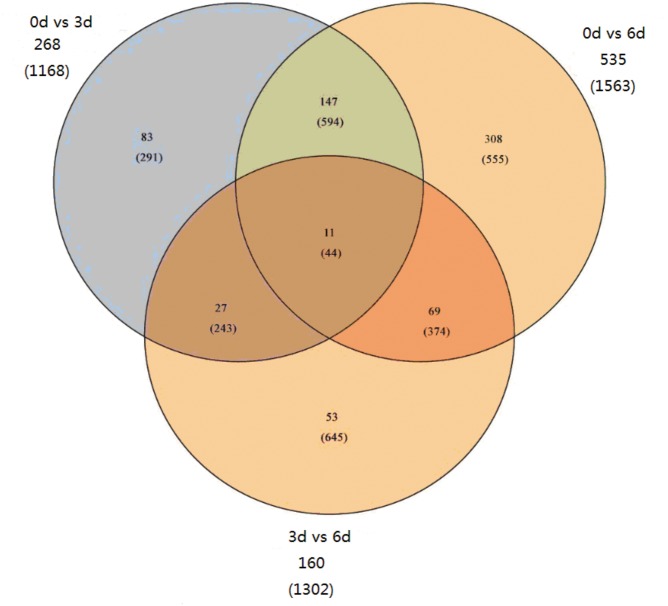
Venn diagram showing the number DEGs for each comparison and the overlaps between three comparison groups. The number in the parenthesis presents the number of isoforms.

We compared our three time points (0, 3, and 6 d) and identified 698 differentially expressed genes (DEGs) (Fig 1, S7 Table) with FDRs of <0.05. We identified 267 DEGs before *Phalaenopsis* explant browning in the comparison of 3 d *vs*. 0 d, 169 (644 isoforms) up-regulated and 98 (523 isoforms) down-regulated. We identified 534 DEGs after explant *Phalaenopsis* browning in the comparison of 6 d *vs*. 0 d, 314 (921 isoforms) up-regulated and 220 (641 isoforms) down-regulated. Finally, we only identified 159 DEGs in the comparison of 6 d *vs*. 3 d, 76 (748 isoforms) up-regulated and 83 (553 isoforms) down-regulated. These results showed that many more genes increased in expression before and after explant browning. Also, the proportion of DEGs was highest in the 0 d *vs*. 6 d comparison and lowest in the 3 d *vs*. 6 d comparison. To identify shared and unique DEGs during explant browning, we generated a Venn diagram based on the three comparisons (Fig 2). Only 11(44 isoforms) DEGs were shared in the three groups.

Furthermore, DEGs that have the best homolog in *Arabidopsis thaliana* and *Oryza sativa* were also studied (S8, S9 and S10 Tables). In the comparison of 3 d vs. 0 d, 6 d vs. 0 d 6 d vs. 3 d, there were 31, 49and 38 isoforms shown sequences identity in *Arabidopsis thaliana*, and 173, 239,173 isoforms hit in *Oryza sativa* respectively. The number of homolog in three groups showed low identity in *Arabidopsis thaliana* comparison with *Oryza sativa*. Based on the homologs in *Arabidopsis thaliana*, we found multiple DEGs involved in biosynthesis of secondary metabolites, nucleotide metabolism, carbohydrate metabolism, and amino acids metabolism, such as: NAD (P)- binding rossmann-fold superfamily protein, nucleoside diphosphate kinase family protein, protein kinase superfamily protein, glucose-6-phosphate dehydrogenase 2, caffeoyl-CoA 3-O-methyltransferase.

The highly expression of DEGs before and after *Phalaenopsis* explant browning was shown in Table 4. The results showed that the expression of pathogenesis-related protein 10c was in higher level and was significantly elevated after explant browning. Notably, glutathione S-transferase (comp16940_0, comp23612_0, comp18470_0, comp23433_0), which involved in Glutathione metabolism, increased by 10–700 times after explant browning, It was also worth mentioning that the expressions of nonsymbiotic hemoglobin (comp23970_0), bibenzyl synthase (chalcone synthase, comp22408_0), 2-hydroxyacyl-CoA lyase (comp22023_0), S-adenosyl-L-methionine synthase (comp19689_0), cytochrome P450 71D11-like (comp20309_0), which appear to be involved in peroxisome, cysteine and methionine metabolism and secondary metabolism, respectively, were very low in the control samples, but increased significantly after *Phalaenopsis* explant browning occurred, the expression increase by 30–600 folds, of which nonsymbiotic hemoglobin expression increase more than 300 folds. These results demonstrated that there is a close relationship between metabolism and browning. The expression of RNA-dependent RNA polymerase (comp23180_c0), was in high level in control explant and was significantly decreased before and after explant browning. Three DEGs involved in energy metabolism, such as V-type proton ATPase 16 kDa proteolipid subunit-like (comp 16560_c0), which take part in oxidative phosphorylation, putative chlorophyll a/b-binding protein (comp 23175_c0), type III chlorophyll a/b-binding protein (comp20543_c0) which relate to photosynthesis expression reduced before and after explant browning, respectively.

**Table 4:** The highly expressed DEGs before and after *Phalaenopsis* explant browning.

gene id	FPKM			Gene Description
	0d	3d	6d	
**Up-regulation**				
comp24400_c0	601.4	13221.09	15931.33	pathogenesis-related protein 10c [Elaeis guineensis]
comp23960_c0	243.1	8625.85	36472.73	gastrodianin-4A [Gastrodia elata]
comp23734_c0	874.74	4548.3	2667.81	lectin [Cymbidium hybrid cultivar]
comp16940_c0	48.41	2552.83	1778.27	tau glutathione S-transferase [Allium cepa]
comp4775_c0	58.2	2416.77	253.37	S-like RNase[Triticum aestivum]
comp22420_c0	195.86	1665.28	1454.83	PREDICTED: formate dehydrogenase, mitochondrial [Vitis vinifera]
comp24492_c0	430.54	1643.42	332.69	RecName: Full = Probable linoleate 9S-lipoxygenase 5; AltName: Full = Leaf lipoxygenase lipoxygenase [Solanum tuberosum]
comp22045_c0	45.2	836.7	672.12	hypothetical protein SORBIDRAFT_04g034160 [Prunus persica]
comp24644_c0	47.75	660.62	319.99	PREDICTED: L-ascorbate peroxidase 2, cytosolic [Vitis vinifera]
comp18704_c0	67.83	641.47	2141.54	phi class glutathione transferase GSTF7 [Populus trichocarpa]
comp22866_c0	55.1	615.43	265.49	PREDICTED: ferritin-3, chloroplastic [Vitis vinifera]
comp23433_c0	0.84	610.48	630.83	glutathione-S-transferase Cla47 [Elaeis guineensis]
comp23612_c0	11.87	597.78	511.05	tau glutathione S-transferase [Allium cepa]
comp19147_c0	124.53	622.35	13177.75	pathogenesis-related protein 10c [Elaeis guineensis]
comp23970_c0	1.54	483.33	1032.45	nonsymbiotic hemoglobin [Raphanus sativus]
comp22408_c0	1.21	25.66	692.42	bibenzyl synthase [Phalaenopsis sp. chalcone synthase]
comp22023_c0	17.61	217.69	655.67	PREDICTED: 2-hydroxyacyl-CoA lyase [Vitis vinifera]
comp22263_c0	131.9	101.1	611.8	RecName: Full = Adenosylhomocysteinase; Short = AdoHcyase; AltName: Full = S-adenosyl-L-homocysteine hydrolase S-adenosylhomocysteine hydrolase
comp23263_c0	41.16	101.1	595.65	acyl CoA ligase [Linum usitatissimum]
comp20309_c0	3.53	46.43	528.32	PREDICTED: cytochrome P450 71D11-like [Glycine max]
comp19689_c0	8.72	80.93	519.67	S-adenosyl-L-methionine synthase [Musa acuminata AAA Group]
comp19151_c0	91.85	403.39	506.52	glutathione S-transferase [Elaeis guineensis]
**Down-regulation**				
comp14578_c0	5435.85	2150.44	459.24	putative chlorophyll a/b-binding protein [*Phalaenopsis* hybrid cultivar]
comp24412_c0	3644.04	297.48	192.06	ORF170[*Phalaenopsis* aphrodite subsp. formosana]
comp24467_c0	2403.94	725.68	240.26	PREDICTED: uncharacterized protein LOC100244671 [Vitis vinifera]
comp19366_c0	1852.48	556.8	382.63	hypothetical protein SORBIDRAFT_07g023340 [Sorghum bicolor]
comp24193_c0	1682.55	38.19	34.45	PREDICTED: BTB/POZ and TAZ domain-containing protein 2-like[Glycine max]
comp18801_c0	1226.98	360.29	72.21	type 1 non-specific lipid transfer protein precursor [Triticum aestivum]
comp16560_c0	1059.9	352.26	190.68	PREDICTED: V-type proton ATPase 16 kDa proteolipid subunit-like [Glycine max]
comp20543_c0	976.56	303.2	175.84	type III chlorophyll a/b-binding protein [Lycoris aurea]
comp23175_c0	873.94	122.29	96.53	RecName: Full = Pyruvate, phosphate dikinase, chloroplastic; AltName: Full = Pyruvate, orthophosphate dikinase; Flags: Precursor pyruvate,ortho
comp21796_c0	764.9	259.27	134.58	Os11g0171300[Oryza sativa Japonica Group]
comp24776_c0	713.77	138.06	117.99	Mitochondrial protein, putative [Medicago truncatula]
comp20608_c0	638.52	123.21	180.07	PREDICTED: dnaJ protein homolog 2 [Vitis vinifera]
comp20205_c1	542.53	428.03	113.87	uncharacterized protein LOC100527231 [Glycine max]
comp17995_c0	541.63	295.51	51.11	HAP3-like protein [Citrus sinensis]
comp15000_c1	531.85	71.35	60.87	PREDICTED: 60S ribosomal protein L51, mitochondrial-like [Glycine max]
comp21138_c0	511.4	819.76	104.11	cationic peroxidase 2 precursor [Glycine max]

The gene were selected at the highest of the three FPKM >500 and were stored by p-value<0.05.

To identified DEGs that were induced before and after *Phalaenopsis* explant browning, the result showed that there were 36 and 69 DEGs induced before and after *Phalaenopsis* explant browning, respectively (S11 Table). The top 10 DEGs were showed in Tables 5 and 6. We found that cytokinin oxidase(comp21576), probable mannitol dehydrogenase(comp21741),4-coumarate:CoA ligase(comp20466), which involved in zeatin biosynthesis, phenylpropanoid biosynthesis, fatty acid elongation, respectively, were highly induced before and after *Phalaenopsis* explant browning.

**Table 5:** The TOP10 of DEGs induced before *Phalaenopsis* explant browning.

Gene id	FPKM		Gene Description
	0d	3d	
comp24050_c0	0	179.85	PREDICTED: pentatricopeptide repeat-containing protein At4g02750 [Vitis vinifera]
comp20452_c0	0	111.27	hypothetical protein SORBIDRAFT_ 07g023110 [Sorghum bicolor]
comp21576_c0	0	108.91	cytokinin oxidase [Dendrobium hybrid cultivar]
comp15076_c0	0	61.13	trypsin inhibitor 3 [Oncidium Gower Ramsey]
comp21640_c0	0	60.97	Peroxidase 52 precursor, putative [Ricinus communis]
comp21741_c0	0	46.64	PREDICTED: probable mannitol dehydrogenase [Vitis vinifera]
comp19746_c0	0	38.15	RecName: Full = Probable mannitol dehydrogenase; AltName: Full = NAD-dependent mannitol cinnamyl alcohol dehydrogenase [Fragaria]
comp20287_c0	0	36.41	PREDICTED: uncharacterized protein LOC100243770 [Vitis vinifera]
comp11152_c0	0	34.58	putative UDP-glucose dehydrogenase 1 [Nicotiana tabacum]
comp18523_c0	0	33.57	extensin-like protein [Vigna unguiculata]

The gene were selected at p-value<0.05

**Table 6:** The TOP10 of DEGs induced after *Phalaenopsis* explant browning.

Gene id	FPKM		Gene Description
	0d	6d	
comp24050_c0	0	274.5	PREDICTED: pentatricopeptide repeat-containing protein At4g02750 [Vitis vinifera]
comp20466_c0	0	184.49	4-coumarate:CoA ligase [Petunia x hybrida]
comp13345_c0	0	147.5	MYB transcription factor MYB92 [Elaeis guineensis]
comp18691_c1	0	144.65	trans-2-enoyl-CoA reductase [Phalaenopsis amabilis]
comp21576_c0	0	127.96	cytokinin oxidase [Dendrobium hybrid cultivar]
comp21741_c0	0	123.9	PREDICTED: probable mannitol dehydrogenase [Vitis vinifera]
comp20452_c0	0	102.5	hypothetical protein SORBIDRAFT_07g023110 [Sorghum bicolor]
comp22222_c0	0	70.1	PREDICTED: probable serine/threonine-protein kinase At1g18390 [Vitis vinifera]
comp29276_c0	0	68.39	glycolate oxidase
comp20432_c1	0	65.15	hypothetical protein slr1753 [Synechocystis sp. PCC 6803]

The gene were selected at p-value<0.05

### Gene ontology (GO) analysis for *Phalaenopsis* transcriptome and DEGs

**Fig 3:**
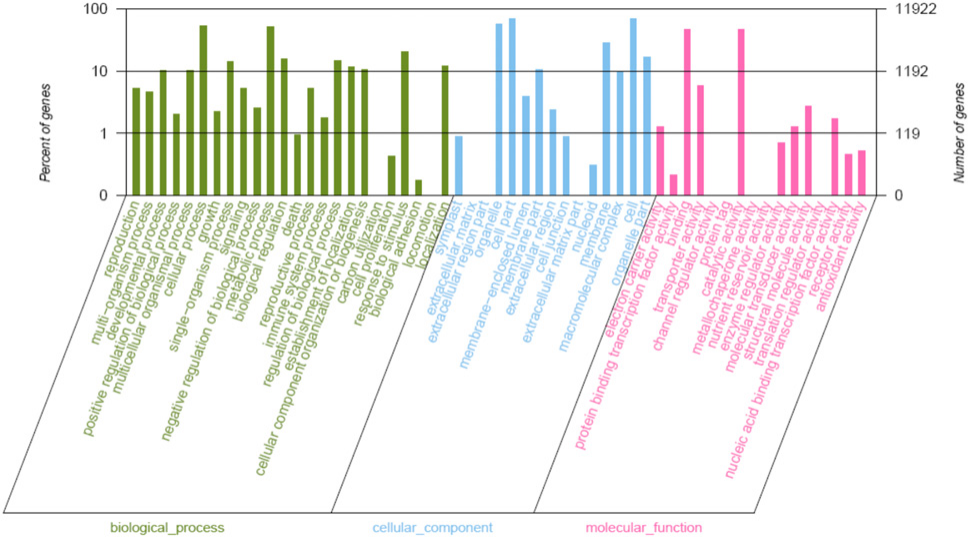
Distribution of the GO categories assigned to the *Phalaenopsis* transcriptome. 21, 384 genes were grouped into 45 sub-categories, which were divided into three categories: cellular component, molecular function, biological process.

To characterize the *Phalaenopsis* transcriptome, we used GO assignments to classify the functions of the predicted *Phalaenopsis* genes. Using Blast2GO, we grouped the 21,384 annotated sequences into 45 sub-categories within the three main GO categories of biological process, cellular component and molecular function (Fig 3). Based on the biological processes, we established 15 categories. We found 6,318 genes (52.99%) involved in cellular processes, 6,167 genes (51.72%) in metabolic processes and 2, 420 genes (20.29%) in responding to stimulus. In addition, we found 14 clusters of genes with roles in cell formation, including cell parts (8,275 genes, 69.41%), organelles (6,885 genes, 57.75%), and membrane (3,431 genes, 28.79%). Based on their putative molecular functions, we also found 23 categories, including genes involved in catalytic activity (5,642 genes, 47.32%) and binding activity (5, 534 genes, 46.42%).

Once we had characterized the transcriptome, we next assigned GO categories to the DEGs and compared them with those in the transcriptome. We found a similar distribution of GO-annotation function of DEGs in the three comparisons (S3 Fig). For biological processes, before and after explant browning, most DEGs were assigned to metabolic process, followed by cellular process and responding to stimulus. For cellular component, the top three categories for DEGs were cell part, cell, and organelles. For molecular functions, the top categories for DEGs were catalytic activity and binding.

**Fig 4:**
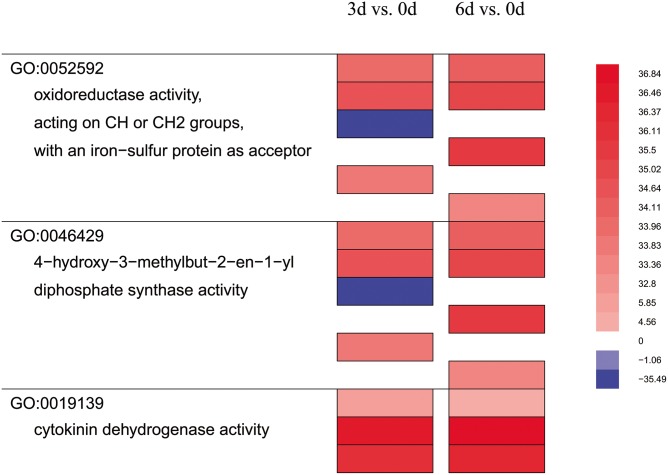
Enriched functional categories in each of the pair-wise comparison. Each vertical column represented the genes that were dramatically up- (red) or down- regulated (blue) comparing the two groups indicated at the top. Lines to the left showed functional category and GO number. Each color bar represents an individual locus.

Furthermore, GO enrichment analyses showed that before *Phalaenopsis* explant browning, the DEGs for 3 d *vs*. 0 d mainly function in the protein–DNA complex, and nucleosome organization. However, after *Phalaenopsis* explant browning, the DEGs for 6 d *vs*. 0 d mainly function in metabolic processes, such as small molecule metabolism, oxoacid metabolism, and carboxylic acid metabolism (S12 Table). We also analyzed the GO enriched functional categories in each of the three pairwise comparisons, which condensed and compressed the genes by removing categories that did not show as significantly different, and uses a false color heat-map to indicate up- or down-regulated classes (Fig 4). Genes encoding enzymes for cytokinin dehydrogenase, oxidoreductase, acting on CH or CH2 groups, with an iron-sulfur protein as acceptor, 4-hydroxy-3-methylbut-2-en-1-yl diphosphate synthase activity were strongly up-regulated in 3 d and 6 d explants compared to control (0 d) (Fig 4). Also, we found no significant change for 6 d *vs*. 3 d in GO function enrichment analyses.

### Pathway analysis for *Phalaenopsis* transcriptome and DEGs

**Fig 5:**
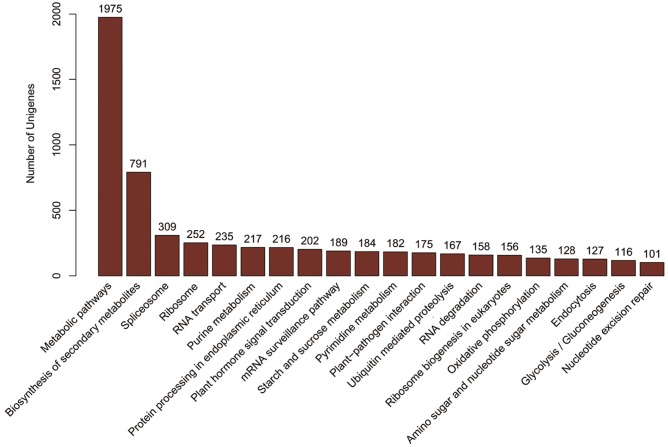
Distribution of *Phalaenopsis* transcriptome sequences among KEGG (Kyoto Encyclopedia of Genes and Genomes) pathways. The top 20 most highly represented pathways are shown. Analysis was performed using Blast2GO and the KEGG database.

**Fig 6:**
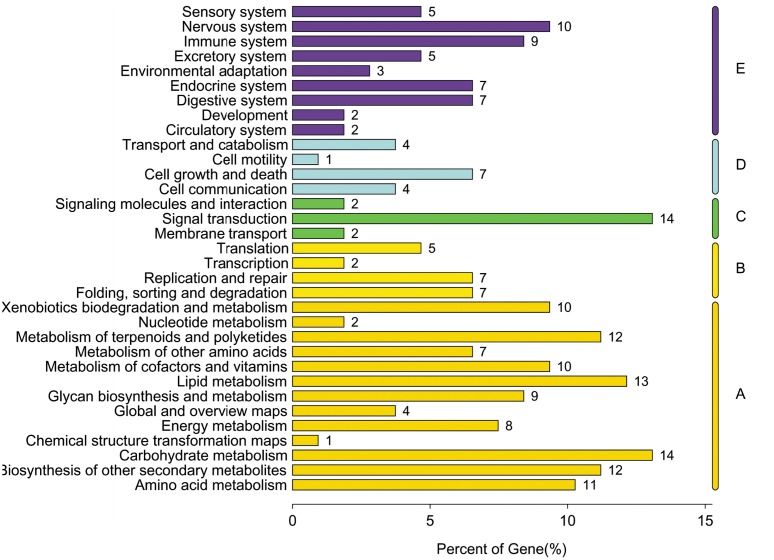
Classification of *Phalaenopsis* genes involved in KEGG pathways. (A: Metabolism, B: Genetic Information Processing, C: Environmental Information Processing, D: Cellular Processes, E: Organismal Systems)

**Fig 7:**
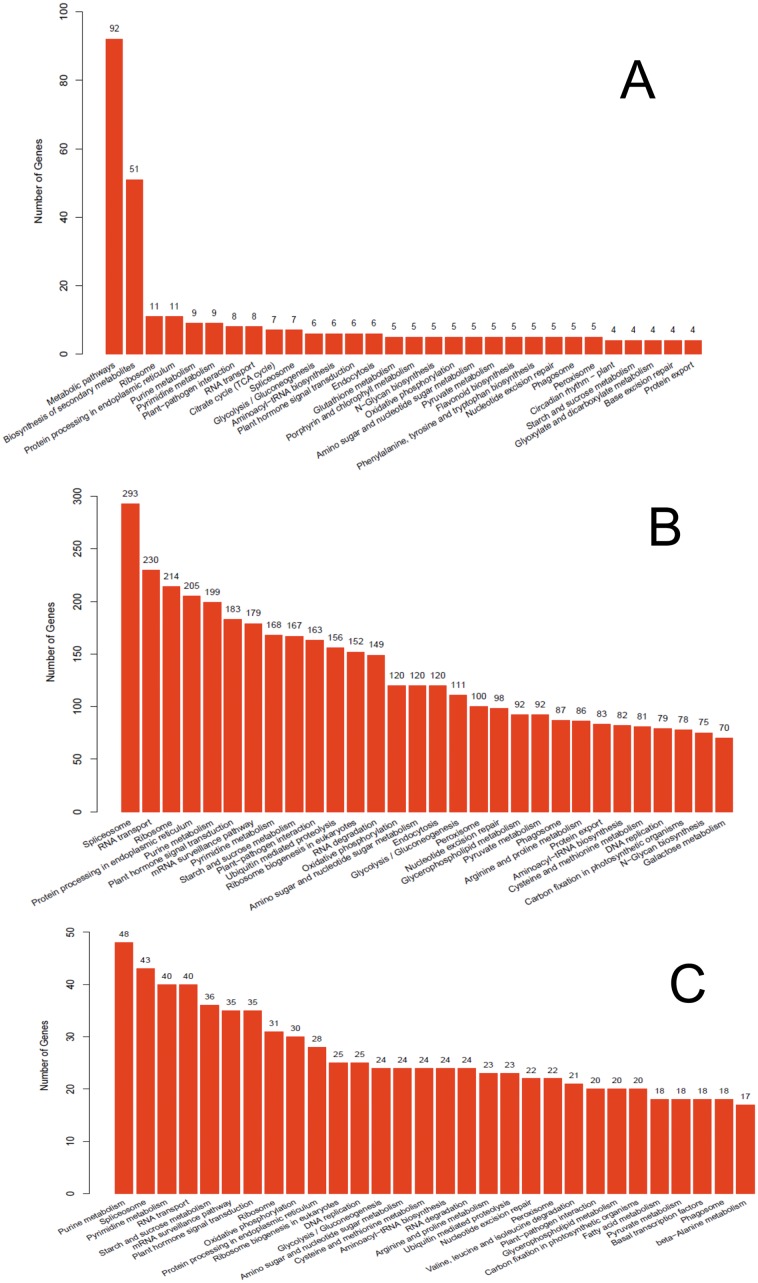
Distribution of *Phalaenopsis* genes and distribution of *Phalaenopsis* genes mapped in mapped in *Arabidopsis thaliana* and *Oryza sativa* among KEGG pathways. The top 20 most highly represented pathways are shown. A: *Phalaenopsis* genes mapped in *Arabidopsis thaliana*, B: Distribution of *Phalaenopsis* genes in KEGG pathways, C: *Phalaenopsis* genes mapped in *Oryza sativa*

Pathway-based analysis can help elucidate the biological functions of genes. To identify the biological pathways that are active in *Phalaenopsis*, we mapped all the unigenes to the reference canonical pathways in KEGG. A total of 14,099 unigenes were assigned to 123 KEGG pathways (S13 Table), where ‘Metabolic pathways’ (1,975 unigenes, 14%) is dominant, followed by ‘Biosynthesis of secondary metabolites ’(791 unigenes, 5%) and ‘Spliceosome ’(309 unigenes, 2%) (Fig 5). KEGG classification analysis identified transcripts involved in signal transduction, carbohydrate metabolism and lipid metabolism have a high degree in *Phalaenopsis* (Fig 6). The distribution of KEGG pathway of *Phalaenopsis* genes was shown in Fig 7. The top 30 pathways of *Phalaenopsis* transcripts mapped to the *Arabidopsis thaliana* and *Oryza sativa* KEGG were showed that the purine metabolism, spliceosome, pyrimidine metabolism and RNA transport were high hit in *Oryza sativa*, while metabolic pathways and biosynthesis of secondary metabolites were high mapped in *Arabidopsis thaliana*. About 293,230 and 214 transcripts mapped in the spliceosome, RNA transport and ribosome in *Phalaenopsis* were tooken over the top 3 (Fig 7).

To characterize the functional consequences of gene expression changes associated with explant browning, we performed pathway enrichment analysis on DEGs based on the KEGG database with p <0.05 as the threshold. We found that 18 of 90 pathways demonstrated significant changes in 3 d *vs*. 0 d, 25/102 pathways in 6 d *vs*. 0 d, and 11/98 pathways in 6 d *vs*. 3 d (S14 Table). Comparison of the three groups of DEGs revealed that phenylpropanoid biosynthesis, flavonoid biosynthesis, phenylalanine metabolism and terpenoid backbone biosynthesis demonstrated significant changes in all three comparisons. There were seven pathways of carbohydrate metabolism, seven pathways of amino acid metabolism, five of lipid metabolism, four pathways of secondary metabolism significantly changed before and after *Phalaenopsis* explant browning (Table 7).

**Table 7:** List of important KEGG pathways enrichment analysis of DEGs before and after *Phalaenopsis* explant browning.

Pathway Id	Pathway name	Background.number	Sample number	
			3d vs.0d	6d vs.0d
**Carbohydrate metabolism**				
ko00010	Glycolysis / Gluconeogenesis	116	12	
ko00040	Pentose and glucuronate interconversions	46	6	
ko00620	Pyruvate metabolism	93	10	
ko00640	Propanoate metabolism	41	6	
ko00630	Glyoxylate and dicarboxylate metabolism	55		10
ko00030	Pentose phosphate pathway	55		11
ko00051	Fructose and mannose metabolism	67		12
**Amino acid metabolism**				
ko00250	Alanine, aspartate and glutamate metabolism	52		9
ko00270	Cysteine and methionine metabolism	84		14
ko00360	Phenylalanine metabolism	50		13
ko00400	Phenylalanine, tyrosine and tryptophan biosynthesis	49		8
ko00480	Glutathione metabolism	67	10	
ko00460	Cyanoamino acid metabolism	30	7	
ko00350	Tyrosine metabolism	34	5	
**Lipid metabolism**				
ko00564	Glycerophospholipid metabolism	97	10	
ko00565	Ether lipid metabolism	28	5	
ko00062	Fatty acid elongation	37		9
ko00071	Fatty acid metabolism	66		13
ko00592	alpha-Linolenic acid metabolism	62		13
**Energy metabolism**				
ko00910	Nitrogen metabolism	41	17	10
ko00195	Photosynthesis	71		17
ko00710	Carbon fixation in photosynthetic organisms	83		16
**Biosynthesis of other secondary metabolites**				
ko00940	Phenylpropanoid biosynthesis	75	16	19
ko00941	Flavonoid biosynthesis	35	7	8
ko00945	Stilbenoid, diarylheptanoid and gingerol biosynthesis	16	4	
ko00960	Tropane, piperidine and pyridine alkaloid biosynthesis	20	4	7
**Translation**				
ko03008	Ribosome biogenesis in eukaryotes	156	15	
ko03010	Ribosome	252	23	
**Signal transduction**				
ko04075	Plant hormone signal transduction	202	18	
**Metabolism of terpenoids and polyketides**				
ko00900	Terpenoid backbone biosynthesis	46	7	9
ko00903	Limonene and pinene degradation	25		5
ko00908	Zeatin biosynthesis	13	3	4
**Transport and catabolism**				
ko04146	Peroxisome	100		14

Pathway enrichment of DEGs was analysis at P-value<0.05.

### Protein domain analysis for *Phalaenopsis* transcriptome and DEGs

Conserved protein domains were identified in the *Phalaenopsis* unigenes against the Pfam database by using Hmmscan software with cutoff E-value≤0.1, 12,341 of 26,565 assembled unigenes matched entries that are corresponding to 3,021 different domains/families (S15 Table). The 20 most abundant domains identified are provided in Table 7. The top 3 abundant domains were identified included Pkinase (protein kinase domain) with 568 unigenes, followed by Pkinase_Tyr (protein tyrosine kinase) with 284 unigenes and RRM_1(RNA recognition motif) with 202 unigenes. The PPR domain proteins and cytochrome P450 families were also highly represented.

**Fig 8:**
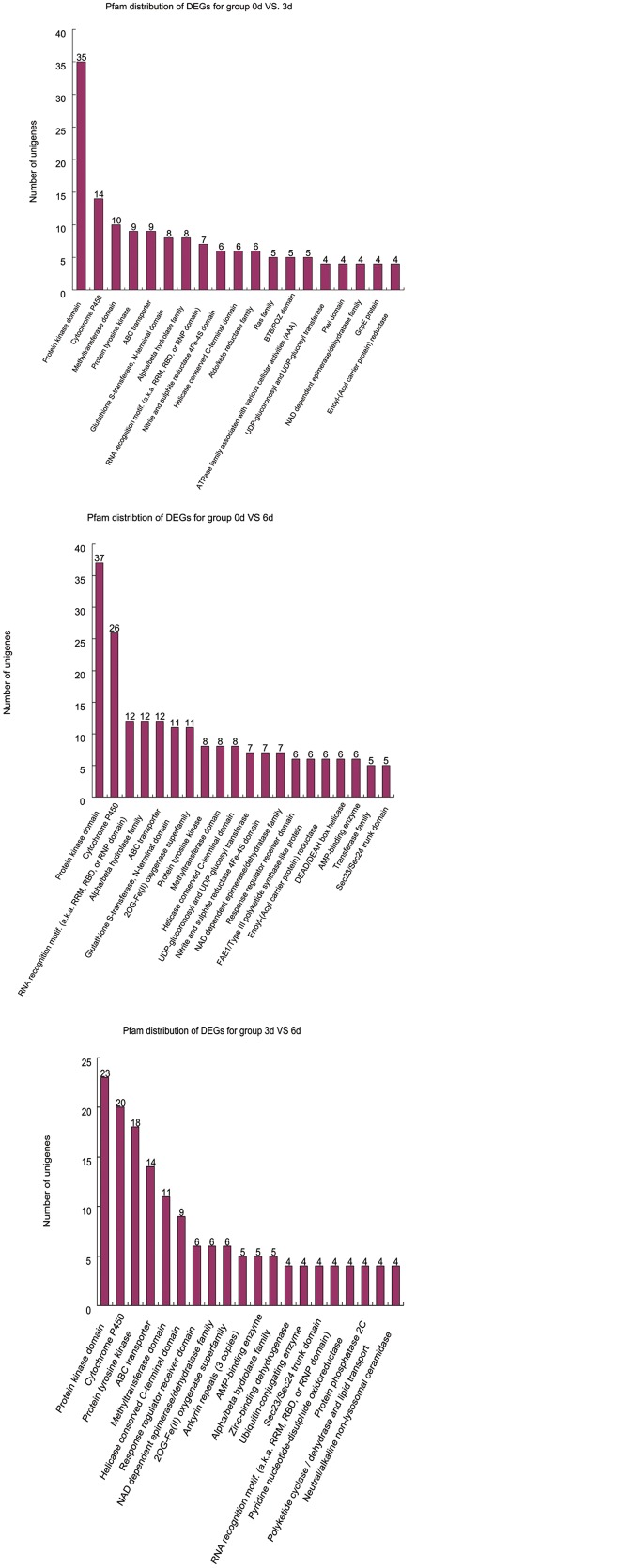
Distribution of the top 20 Pfam domains identified in three groups of DEGs.

To specifically identify DEGs protein domains in the Pfam database, comparison of Pfam searches between three groups of DEGs indicated an overall similarity of identified domains. The top 20 domains were shown in Fig 8. Protein kinase domain, cytochrome P450, methyltransferase domain, Protein tyrosine kinase and ABC transporter were most highly represented in three groups of DEGs.

### Validation of the expression profile data using real-time PCR

**Fig 9:**
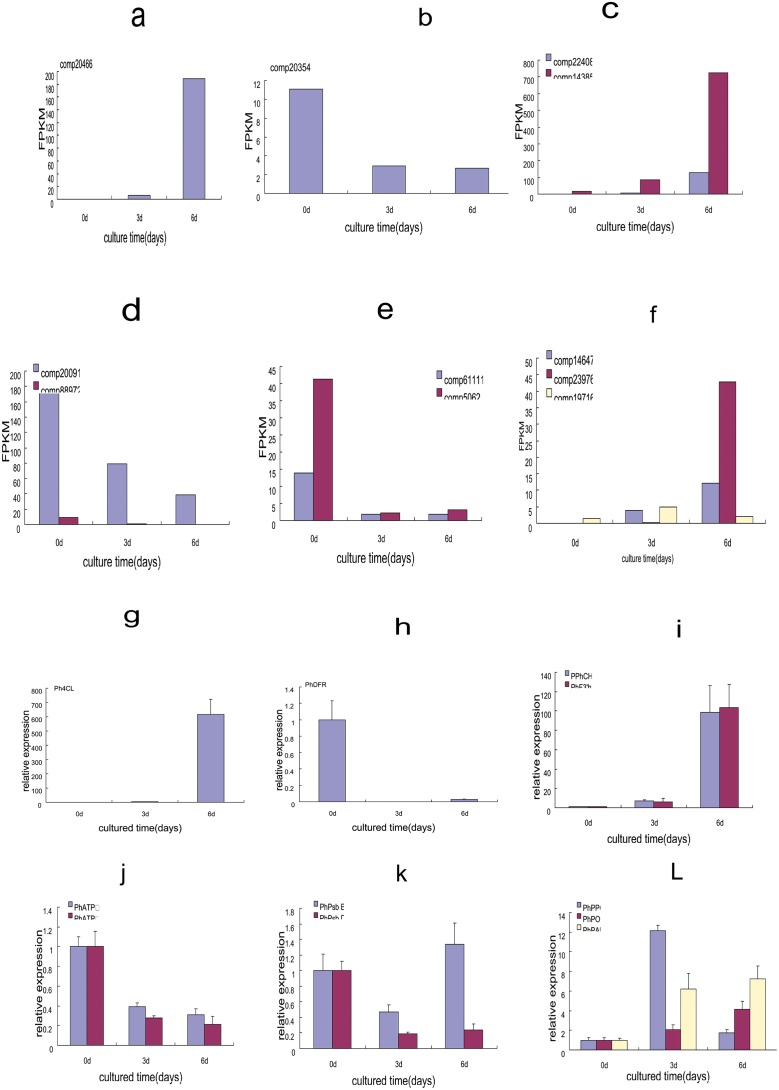
Transcript profile(a-f) and qPCR (g-l) results of selected genes during *Phalaenopsis* explant browning. The genes and transcriptome number: comp 20466 (*4CL*), comp 20354 (*DFR*), comp 22408 (*CH*S), comp 14385 (*F3'H*), comp 20091 (*ATP* γ subunite), comp 88972 (*ATP* α subunite); comp 61111 (*psbB*), comp 5062 (*psbD*), comp 14647 (*POD*), comp 23976 (*PAL*), comp 19716 (*PPO*). The left y-axis indicates the FPKM (fragments per kilobase of exon modle per millions mapped reads). In a-f and the relative expression level by qPCR(2ΔΔ ct), is presented as fold- changes in gene expression normalized to the actin gene in g-l.

Based on KEGG pathway enrichment analysis and previous studies [4–6, 11–12], we selected eleven DEGs and subjected them to quantitative real-time PCR (qRT-PCR) to validate the results of the expression profile analysis (Fig 9). We found a high correlation (R^2^ = 0.8841) between RNA-seq and qRT-PCR results (S1 Table). For example, the transcript levels of *CHS*(chalcone synthase), *4CL*(4-coumaroyi·CoA ligasc), and *F3’H* (flavonoid3’-hydroxylase)were high in 6 d explants, and *DFR* (dihy- droflavonol 4-reductase)decreased in 3 and 6 d explants (Fig 9h and 9i). *ATPase* γ subunit, *ATPase* α subunit, *psbB*, and *psbD* were down-regulated in the 3 and 6 d explants, consistent with the results of expression profile analysis. Only the *psbB* transcript level increased in the 6 d explants, in contrast to the sequence data (Fig 9e and 9k). As expected [4, 11], the transcripts of *PPO*, *POD*, and *PAL* were up-regulated during browning (Fig 9I). Our results indicate that our RNA-seq data reliably identified browning-related genes in *Phalaenopsis*.

## Discussion

### Transcriptome and expression profile analysis of DEGs during *Phalaenopsis* explant browning

Using the Illumina HiSeq 2000 next generation sequencing technology, we obtained transcriptome sequences from explants that were cultured for 0, 3, and 6 d. We assembled more than 79,434,350 reads into approximately 31,708 isogenes and annotated 26,565 proteins. Homology searches showed that 63.78 and 61.71% isogenes have significant similarities to *Oryza sativa* and *Arabidopsis thaliana* protein sequences and about 85.66% of isogenes show identity with the publicly available *Phalaenopsis* equestris. Our data show more sequences with significant identity to known sequences in the plant proteome than the sequences identified by Hsu *et al*. [24]. Also, we annotated and functionally mapped 21, 348 sequences to GO terms. Gene annotation can help predict potential genes and their functions at the transcriptome level. In this study, we found predominant gene clusters involved in the cellular and metabolic processes of biological processes, the binding and catalytic activities of molecular functions, as well as the cell, cell part, and organelles categories of cellular components. Our results were different from those obtained in *Phalaenopsis aphrodite*, in which the identified genes were predominantly involved in the biosynthesis of macromolecules and in nitrogen, protein, and nucleic acid metabolism [20]. This difference may be due to the fact that we constructed our cDNA library only from *Phalaenopsis* leaf tissue.

The DEGs among the explants cultured for 0, 3, and 6 d probably relate to their metabolism and functions. We found that 267 and 534 genes exhibited significant differences in expression before and after (3d and 6d) *Phalaenopsis* explant browning, respectively. Among them 36 and 69 DEGs were induced before and after explant browning, respectively. The three comparisons shared only 11 genes in common, indicating that the patterns of gene expression differed over time during explant browning. The identified DEGs were involved in biological processes, cellular components, and molecular functions GO terms. Pfam search showed that protein kinase domain, cytochrome P450, methyltransferase domain, protein tyrosine kinase and ABC transporter were most highly represented in three groups of DEGs. Protein kinase and its subcategories tyrosine-protein kinase were known to regulate the majority of cellular pathways. Cytochrome P450 is a large plant protein family that might contribute to extensive modifications of various secondary compounds. These results revealed DEGs with multiple functions, suggesting that 3 d and 6 d were critical stages for explant browning, when many substances related to browning were produced.

### DEGs involved in polyphenol synthesis and phenylpropanoid pathways before and after *Phalaenopsis* explant browning

Previous studies indicated that phenol oxidase activity results in explant browning and the degree of explant browning positively correlates with the contents of phenolic compounds [2–5, 25]. The accumulation and oxidation of phenolic compounds causes enzymatic browning. Also, reduction of polyphenol synthesis and inhibition of the phenylpropanoid pathway repressed browning [26–28]. PAL is the first dedicated enzyme in the phenylpropanoid pathway and converts phenylalanine into trans-cinnamic acid, providing a substrate for further synthesis of phenolic compounds. PPO and POD also function in enzymatic browning. Both enzymes have common diphenolic substrates leading to brown products. In our previous studies, we found that tannin filled in the vascular bundles of browning tissues [29]. Based on previous work and this study, we selected seven genes for qPCR: *PhCHS*, *Ph4CL*, *PhF3’H*, and *PhDFR*, which function in flavonoid biosynthesis, and *PhPPO*, *PhPOD*, and *PhPAL*, which function in phenylalanine metabolism, and which also showed significant changes in the KEGG pathway enrichment analysis in this study. Consistent with our expectations, *PhPPO*, *PhPOD*, and *PhPAL* were up-regulated in *Phalaenopsis* explant browning [2, 4, 8, 30] and qRT-PCR confirmed the expression profiling obtained in this study.

Anthocyanins, a class of flavonoids that is responsible for the colors in fruits and most flowers of higher plants, are major water soluble pigments [31,32] and exhibit important physiological functions, such as antioxidative [33,34]. The degradation of anthocyanins and/or the oxidation of phenolics caused by polyphenol oxidase (PPO) results in an enzymatic browning reaction of fruits and vegetables [35,36]. Genes in anthocyanin biosynthesis such as *PhPAL*, *PhCHS*, *Ph4CL*, *PhF3’H* were up-regulated in 6 d explants of *Phalaenopsis* indicates that anthocyanin may play important roles in the early stages of *Phalaenopsis* explant browning. Previous studies have shown that disruptions of oxidation and reduction may induce tissue browning, leading to reactive oxygen species (ROS) bursts, which would affect the membrane integrity [37–39]. ROS which were induced in tissue culture is eliminated by biosynthesis of anthocyanin. DFR is a key enzyme in the catalysis of the stereospecific reduction of dihydroflavonols to leucoanthocyanidins in anthocyanin biosynthesis. Over-expression DFR in the sweet potato enhanced scavenging of reactive oxygen radicals in plants under stressful conditions [40]. *PhDFR* showed opposite expression profile compared to that of *PhPAL*, *Ph4CL*, *PhCHS* and *PhF3’H* in the early stages of *Phalaenopsis* explant, so anthocyanin biosynthesis may be effected. This may lead to imbalance of ROS metabolism and membrane lipid peroxidation, and destroy of cell membrane integrity.

Meanwhile, Cytochrome P450 plays an important role in flavone biosynthesis and has been well characterized. About 115 different cytochrome P450 isogenes were detected in our RAN sequencing data. The cytochrome P450 71D11-like was highly expression after *Phalaenopsis* explant browning (Tables 4 and 8). Cytochrome P450 expressed only after *lotus* tissue browning [41], indicating that browning was beneficial for secondary metabolite production.

**Table 8:** Distribution of the top 20 Pfam domains identified in translated *Phalaenopsis* sequences.

Pfam_entry_name	description of Pfam entry	annotated isogenes number
Pkinase	Protein kinase domain	568
Pkinase_Tyr	Protein tyrosine kinase	284
RRM_1	RNA recognition motif. (a.k.a. RRM, RBD, or RNP domain)	202
PPR_3	Pentatricopeptide repeat domain	138
PPR_2	PPR repeat family	117
p450	Cytochrome P450	115
DEAD	DEAD/DEAH box helicase	88
ABC_tran	ABC transporter	84
Helicase_C	Helicase conserved C-terminal domain	78
PPR_1	PPR repeat	70
AAA	ATPase family associated with various cellular activities (AAA)	66
PP2C	Protein phosphatase 2C	66
Abhydrolase_6	Alpha/beta hydrolase family	57
Abhydrolase_5	Alpha/beta hydrolase family	54
Epimerase	NAD dependent epimerase/dehydratase family	53
Myb_DNA-binding	Myb-like DNA-binding domain	52
TPR_14	Tetratricopeptide repeat	52
Methyltransf_11	Methyltransferase domain	51
2OG-FeII_Oxy	2OG-Fe(II) oxygenase superfamily	47
RRM_6	RNA recognition motif (a.k.a. RRM, RBD, or RNP domain)	47

Analysis was performed using HMMER with Pfam database.

### DEGs involved in photosynthesis, energy synthesis and other metabolism before and after *Phalaenopsis* explant browning

Photosystem II (PSII) is a multi-subunit, pigment-protein complex localised in the chloroplast thylakoid membranes. The PSII complex is composed of a number of proteins including D1 (PsbA), D2 (PsbD), CP43 (PsbC), CP47 (PsbB), apoproteins of cytochrome b559, and the extrinsic manganese- stabilizing protein, along with a number of small proteins [42]. In our KEGG pathway enrichment analysis, we found that DEGs involved in photosynthesis also changes significantly in browning explants, so we selected *PhpsbB*, and *PhpsbD* for qRT-PCR. F-ATPases (also known as F1F0-ATPase, or H(+)-transporting two-sector ATPase) (EC:3.6.3.14) function as the prime producers of ATP in mitochondria, chloroplasts and bacterial plasma membranes, using the proton gradient generated by oxidative phosphorylation (mitochondria) or photosynthesis (chloroplasts). Thus, we also selected the genes encoding the *Ph*ATPase γ subunit, and A*Ph*TPase α subunit for qRT-PCR. Our qRT-PCR results were in accordance with the RNA-seq expression profiles in which the transcript levels decreased in the 3 and 6 d explants. By contrast, our qRT-PCR analysis showed that the transcript levels of *PhpsbB* increased in the 6 d explants. We have found that during explant browning, chloroplasts shrink to dark-colored structures and chlorophyll b content declines [25,29]. In this study, we found that the abundance of *PhpsbB* and *PhpsbD* declined before and after *Phalaenopsis* explant browning. Consistent with this results, two kinds of chlorophyll a/b binding protein were found to be decreased significantly during explant browning(Table 4). To our knowledge, this is the first report to show that photosynthesis genes are associated with explant browning. Liu *et al* [18] also confirmed that photosynthesis changes during pear fruit browning. These metabolic processes will disrupt the energy metabolism providing a molecular context for the formation of ROS in browning tissue. We conceive that the wound-induced natural defense response leads to accumulation of toxic compounds that impair photosynthesis in browning plant tissues and ultimately damage or kill plant cells.

In plant tissues, ATP content is important for regulating anti-oxidase expression, such as superoxide dismutase, catalase, and ascorbate peroxidase [43]. Jiang *et al*. [40] also found that ATPase was differentially expressed in *Lotus* tuber browning tissue using 2-DE and MALDI-TOF-TOF for protein profile analysis. Proteins related to nucleotide metabolism: adenosine kinase, and nucleoside diphosphate kinase were down-expressed after *Lotus* tissue browning. Their down-regulations indicated that substance metabolism was out of control after browning and the abilities of energy synthesis and utilization declined, accompanied by disorder of ATP energy metabolism. Senescence and stress can also lead to lower ATP production and higher accumulation of reactive oxygen species, resulting in membrane system disorder and triggering browning [42]. Moreover, using LCQ MS/MS for protein expression analysis in explants cultured for 3 d, we linked ATPase with explant browning [12]. In line with this, we herein verified that the transcript levels of the genes encoding the *Ph*ATPase γ and *Ph*ATPase α subunits decreased in the 3 d and 6 d explants, implying that *Ph*ATPase has important functions during explant browning. The expression of V-type proton ATPase 16 kDa proteolipid subunit-like also decreased significantly before and after explant browning (Table 4). Down-regulation of genes encoding ATPase subunits indicated that energy synthesis and utilization declined. Less energy means incapacity to repair membranes, in turn, loosening of cellular compartmentalization allows the release of PPOs and phenols to the cytosol, triggering the actual discolouration reactions. Thus, we consider that ATP metabolism might affect explant browning in *Phalaenopsis*.

Before and after *Phalaenopsis* explant browning, DEGs involved in amino acid metabolism, carbohydrate metabolism, lipid metabolism were significantly changed (Table 7). A total of 22 and 44 DEGs involved in amino acid metabolism before and after explant browning, indicating that expressions of some proteins resulted in promotion of the browning reaction. After *Lotus* tissue browning, Jiang *et al* found the expression of proteins relating to amino acid metabolism: cysteine synthase and glutamate-ammonia ligase reduced, respectively [40]. Related to lipid metabolism the expression type 1 non-specific lipid transfer protein reduced during explant browning, implying that lipid might be related to plant stress tolerance. Many DEGs involved in lipid degradation pathways were significantly up-regulated in apple fruit browning disorder [17]. It is understandable that changes in these metabolic processes will disrupt the energy metabolism and cell compartments, resulting oxidation of phenolic compounds and influence the explant browning.

## Conclusions

Our RNA-seq analysis of changes in gene expression substantially increases the number of available equestris in *Phalaenopsis* and provides comprehensive information on the mechanism of *Phalaenopsis* explant browning at a genome-wide scale. Most of the DEGs we identified were up-regulated during *Phalaenopsis* explant browning. More importantly, transcripts of the genes that control secondary metabolism, energy metabolism, carbohydrate metabolism, and lipid metabolism were differentially expressed in explant browning. In addition, in this study, we identified many new browning-related genes. More detailed and extensive analyses of the identified genes will be essential to further decipher their possible roles in explant browning. Thus, our data provide genome-wide information that enables further exploration of explant browning.

## Supporting Information

S1 Fig The figure of cultivar of *Phalaenopsis* used in this study. (EPS)

S2 Fig The picture of explant culture for 3d and 6d showed the degree of explant browning. (EPS)

S3 Fig Functional annotiation based on GO categories of DEGs from three groups in *Phalaenopsis*. Each set of 3 vertical bars (blue, red, yellow) indicated the percent of DEGs in the three categories: molecular functions(A), cellular components (B), and biological processes (C).
(TIF)

S1 Table List of Primers for quantitative real-time PCR. (DOC)

S2 Table Sequence length distrubtion of isogenes of *Phalaenopsis* transcriptome analysis. (DOC)

S3 Table Annotation of *Phalaenopsis* transcriptome. (XLS)

S4 Table Homology analysis of *Phalaenopsis* proteome with *Abrabidopsis thaliana*. (XLS)

S5 Table Homology analysis of *Phalaenopsis* proteome with *Oryza sativa*. (XLS)

S6 Table Three kind of explant FPKM analysis. (XLS)

S7 Table DEGs analysis of three groups. FDR <0.05 and the absolute value of log2Ratio ≤1 were used as the threshold to judge the significance of gene expression difference.
(XLS)

S8 Table DEGs of 0d vs. 3d homolog in *Arabidopsis* and *Oryza*. (XLS)

S9 Table DEGs of 0d vs. 6d homolog in *Arabidopsis* and *Oryza*. (XLS)

S10 Table DEGs of 3d vs. 6d homolog in *Arabidopsis* and *Oryza*. (XLS)

S11 Table DEGs induced before and after *Phalaenopsis* explant browning. (XLSX)

S12 Table GO functional enrichment analysis of DEGs. Fisher’s exact test with FDR <0.05
(DOC)

S13 Table KEGG pathway analysis of *Phalaenopsis* proteome. A totalof 14,099 sequences were assigned to 123 KEGG pathways.
(XLS)

S14 Table KEGG pathway enrichment analysis of DEGs. (XLS)

S15 Table Results of a Pfam domain search using *Phalaenopsis* transcriptomic sequences. (XLS)
