# Supplementary figures and images for: Genome-Wide Transcriptome and Expression Profile Analysis of Phalaenopsis during Explant Browning
Source: PLoS One. 2015 Apr 14;10(4):e0123356. doi: 10.1371/journal.pone.0123356 (PMC4397044; doi:10.1371/journal.pone.0123356)

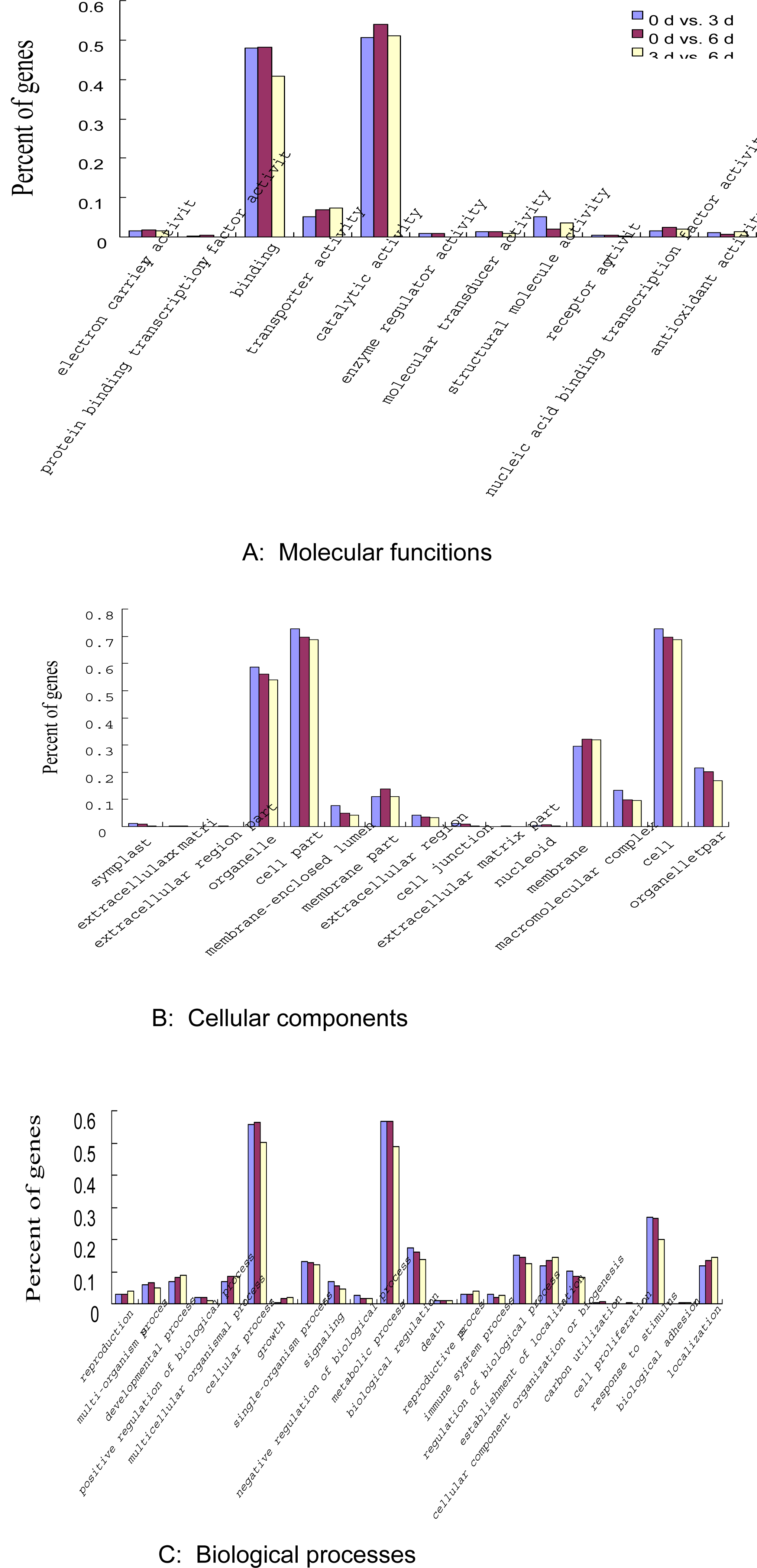

Supplement: S3 Fig — Each set of 3 vertical bars (blue, red, yellow) indicated the percent of DEGs in the three categories: molecular functions(A), cellular components (B), and biological processes (C). (TIF) [file pone.0123356.s003.tif]
