# Supplementary material for: Genome-Wide Transcriptome and Expression Profile Analysis of Phalaenopsis during Explant Browning
Source: PLoS One. 2015 Apr 14;10(4):e0123356. doi: 10.1371/journal.pone.0123356 (PMC4397044; doi:10.1371/journal.pone.0123356)
Supplement: S1 Table — (DOC) [file pone.0123356.s004.doc]

**S1 Table List of Primers for quantitative real-time PCR**

| Gene name | Primer sequences-forward (F) and reverse (R) | The correlation between RNA-seq and qPCR (R^2^) |
| --- | --- | --- |
| 4CL(comp 20466) | F 5' CATCGTCGTCATCAACATATC 3' R 5' GAGGAACGCTGAACTAAAGG 3' | 0.99972 |
| PAL(comp 23976) | F 5' TACAGAAGCCGAAACAGGACC 3' R 5' CAATCAAAGGGTTGTCGTTCA 3' | 0.629376 |
| CHS(comp 22408) | F 5' AGTTCCAATCCGAAATGCC 3' R 5' CGGGCGAGTTTCATCATAG 3' | 0.999868 |
| DFRCcomp 20354) | F 5' GGAGCGACCTCAATTTCATC 3' R 5' ACTTGGCGGCATCTCATCT 3' | 0.9987716 |
| F3’H(comp 14385) | F 5' TTAGGGATTAGGTAGCCGTCG 3' R 5' TTCCAAGCCGTCATCAAAGA 3' | 0.9989692 |
| POD(comp 14647) | F 5' TCAGGCGGAGGACATTATCA 3' R 5' GACAGCGTCTTTCTTGTGGAG 3' | 0.9998855 |
| PPO(comp 19716) | F 5' ATCTTCTTTGCCCACCATTC 3' R 5' CTTCACTTTCACCAGCTTCG 3' | 0.9939039 |
| ATP enzyme α subunite (comp 88972) | F 5' TCATACTTCCTTCACCTAAACG 3' R 5' TTCCAAACAGGCACAAGC 3' | 0.9992004 |
| ATP enzyme γ subunite (comp 20091) | F 5' CAATCTCCAAAATCTCACCAG 3' R 5' GCTTTGCTTCCCCTGTATC 3' | 0.9810593 |
| psbB(comp 61111) | F 5' ATTCCATCTTAGTGTCCGTCC 3' R 5' CGATTGGGGTAGTTGCTGA 3' | 0.1250975 |
| psbD(comp 5062) | F 5' TACTATGGGGTCCTGAAGCA 3' R 5' GCAATTGAACAGAACGAGC 3' | 0.9995291 |
| Actin | F 5' TGTAAGGGACGTGAAGGAGAAG 3'R 5' GGTCATTGATGGCTGGAAGAG 3' |  |
