# Supplementary material for: Genome-Wide Transcriptome and Expression Profile Analysis of Phalaenopsis during Explant Browning
Source: PLoS One. 2015 Apr 14;10(4):e0123356. doi: 10.1371/journal.pone.0123356 (PMC4397044; doi:10.1371/journal.pone.0123356)
Supplement: S2 Table — (DOC) [file pone.0123356.s005.doc]

**S2 Table Sequence length distrubtion of isogenes of *Phalaenopsis*** transcriptome analysis

| Length of isogene | Number of isogene | Ratio(%) |
| --- | --- | --- |
| 1-400 | 2,649 | 8.35% |
| 401-600 | 6,860 | 21.63% |
| 601-800 | 4,626 | 14.59% |
| 801-1000 | 3,584 | 11.30% |
| 1001-1200 | 3,090 | 9.75% |
| **… … …** |  |  |
| ALL | 31,708 | 100.00% |
