# Supplementary material for: Genome-Wide Transcriptome and Expression Profile Analysis of Phalaenopsis during Explant Browning
Source: PLoS One. 2015 Apr 14;10(4):e0123356. doi: 10.1371/journal.pone.0123356 (PMC4397044; doi:10.1371/journal.pone.0123356)
Supplement: S12 Table — Fisher’s exact test with FDR <0.05 (DOC) [file pone.0123356.s015.doc]

**S12 Table GO functional enrichment analysis of DEGs** (Fisher’s exact test with FDR ﹤0.05)

|  | id | description | p_fdr |
| --- | --- | --- | --- |
| 0d vs.3d | GO:0000786 | nucleosome | 0 |
|  | GO:0032993 | protein-DNA complex | 0 |
|  | GO:0034728 | nucleosome organization | 0.002 |
|  | GO:0006334 | nucleosome assembly | 0.002 |
|  | GO:0065004 | protein-DNA complex assembly | 0.002 |
|  | GO:0071824 | protein-DNA complex subunit organization | 0.002 |
|  | GO:0046982 | protein heterodimerization activity | 0.009 |
|  | GO:0016725 | oxidoreductase activity, acting on CH or CH2 groups | 0.014 |
|  | GO:0045652 | regulation of megakaryocyte differentiation | 0.021 |
|  | GO:0045653 | negative regulation of megakaryocyte differentiation | 0.021 |
|  | GO:0045638 | negative regulation of myeloid cell differentiation | 0.021 |
|  | GO:0045637 | regulation of myeloid cell differentiation | 0.021 |
|  | GO:0052592 | oxidoreductase activity, acting on CH or CH2 groups, with an iron-sulfur protein as acceptor | 0.021 |
|  | GO:0046429 | 4-hydroxy-3-methylbut-2-en-1-yl diphosphate synthase activity | 0.021 |
|  | GO:0044427 | chromosomal part | 0.026 |
| 0d vs 6d | GO:0044281 | small molecule metabolic process | 0.002 |
|  | GO:0043436 | oxoacid metabolic process | 0.006 |
|  | GO:0019752 | carboxylic acid metabolic process | 0.006 |
|  | GO:0006082 | organic acid metabolic process | 0.006 |
|  | GO:0042180 | cellular ketone metabolic process | 0.008 |
|  | GO:0006732 | coenzyme metabolic process | 0.008 |
|  | GO:0016746 | transferase activity, transferring acyl groups | 0.009 |
|  | GO:0009108 | coenzyme biosynthetic process | 0.013 |
|  | GO:0016747 | transferase activity, transferring acyl groups other than amino-acyl groups | 0.013 |
|  | GO:0006002 | fructose 6-phosphate metabolic process | 0.014 |
